# Abnormal carbohydrate metabolism in a canine model for muscular dystrophy

**DOI:** 10.1017/jns.2017.59

**Published:** 2017-11-23

**Authors:** Andressa R. Amaral, Márcio A. Brunetto, Marina P. Brólio, Daniela S. Cima, Maria A. Miglino, João Paulo F. Santos, Carlos E. Ambrósio

**Affiliations:** 1 Veterinary Medicine Department, Faculty of Animal Science and Food Engineering (FZEA), São Paulo University (USP), Av. Duque de Caxias Norte, 225, Pirassununga, 13635-900, SP, Brazil; 2 Animal Nutrition and Production Department, Faculty of Veterinary Medicine and Animal Science (FMVZ), University of São Paulo (USP), Av. Duque de Caxias Norte, 225, Pirassununga, 13635-900, SP, Brazil; 3 Surgery Department, Faculty of Veterinary Medicine and Animal Science (FMVZ), University of São Paulo (USP), Av. Prof. Dr Orlando Marques de Paiva, 87, São Paulo, 05508-270, SP, Brazil; 4 Veterinary Anesthesiology Department, Guarulhos University, Anton Philips Street, 1, Vila Hermínia, Guarulhos, 07030-010, SP, Brazil

**Keywords:** Glucose, Insulin, Golden retriever muscular dystrophy, Homeostasis model assessment index, DMD, Duchenne muscular dystrophy, GRMD, golden retriever muscular dystrophy, HOMA, homeostasis model assessment, IR, insulin resistance

## Abstract

The canine golden retriever muscular dystrophy (GRMD) model is the best animal model for studying Duchenne muscular dystrophy in humans. Considering the importance of glucose metabolism in the muscles, the existence of metabolic and endocrine alterations in a wide range of muscular dystrophies, and the pre-existing relationship between blood insulin concentration and muscular atrophy, the present study aimed to evaluate the postprandial glucose and insulin response in GRMD dogs. A total of eighteen golden retriever dogs were randomly distributed into three experimental groups: healthy/control (G1), female GRMD carriers (G2), and male dogs affected by GRMD (G3). Higher plasma resting glucose levels (*P* = 0·0047) were seen in G2 and G3 compared with G1, as was the case for minimum (*P* = <0·0001), mean (*P* = 0·0002) and maximum (*P* = 0·0359) glucose values for G3 compared with G1. Fructosamine concentrations were in accordance with reference values found in the literature for dogs. Insulin levels were lower in G3 compared with G1 (*P* = 0·0065); however, there was no evidence of insulin resistance according to the homeostasis model assessment index values obtained. As for the evaluation of postprandial responses, fluctuations of glucose (*P* = 0·0007) and insulin (*P* = 0·0149) were observed in G1 and G2, while in G3 the values remained constant. The results allowed us to identify metabolic changes related to carbohydrate metabolism in GRMD dogs, highlighting the importance of adequate food management for these animals.

Duchenne muscular dystrophy (DMD) is the commonest form of muscular dystrophy in humans^(^^1^^)^ and it is inherited from a recessive X-linked gene, affecting 1 in 3500 newborn males^(^^2^^)^. The disease involves progressive muscle degeneration until death at about 20 years of age, usually caused by cardiorespiratory events^(^^3^^)^. For researching new therapies to improve the quality of life or discover an effective treatment for those affected by this disease, the use of animal models has major importance^(^^4^^)^.

There are many animal models for studying DMD, among them, the MDX mouse and the golden retriever muscular dystrophy (GRMD) dog; however, the GRMD dog is the most appropriate^(^^5^^)^ because, like in humans, it is a spontaneously occurring disease that progressively causes degeneration of muscle fibres^(^^6^^)^.

Additionally, adult GRMD dogs show phenotypic characteristics similar to boys affected by DMD, such as decreased body weight and muscle quantity^(^^7^^)^, differently from the mice, where the phenotype is different from the human phenotype of the same disease^(^^8^^)^.

Muscle atrophy is frequently observed in patients with insulin deficiency, which leads to decreased rate of protein synthesis in skeletal muscle^(^^9^^,^^10^^)^. Skeletal muscle is also responsible for approximately 40 % of the total body mass and plays a major role in energy balance, since it stimulates the uptake, disposal and storage of glucose^(^^11^^)^.

Glucose is the main energy source for cells, which may be obtained directly from the food or converted from other metabolites through gluconeogenesis^(^^12^^)^. In single-stomached animals like dogs, dietary starches are hydrolysed enzymically in the gastrointestinal tract and the resulting glucose is absorbed and transported to the liver where the processes of glycolysis (postprandial conditions) or gluconeogenesis (fasting conditions) occur. The metabolic activities of this tissue, along with the hormonal signalling of glucagon and insulin released by α and β cells of pancreas, respectively, effectively regulate blood glucose homeostasis^(^^13^^)^.

The values of plasma glucose concentrations are used as indicators of carbohydrate metabolism and reflect the balance between glucose production and uptake^(^^14^^)^. Fructosamine is a glycated plasma protein that can be used as a carbohydrate metabolism indicator since it reflects the glycaemic behaviour beyond that observed by plasma glucose at the time of collection^(^^15^^)^.

Insulin is a multifunctional hormone. It is related to the anabolic and anticatabolic processes in mammals^(^^16^^)^ and plays two other important functions: stimulation of carbohydrate and lipid metabolism for induction of cellular enzymes, especially in hepatocytes, and transportation of glucose across plasma membranes of insulin-sensitive cells, mainly in fat cells and skeletal muscle.

The homeostasis model assessment (HOMA) index, a widely used tool in human medicine as an indicator of insulin sensitivity, can properly reflect how animals metabolise their diet. Evaluating the results obtained by this index enables possible correction of food management for these animals. Previous studies in dogs have already used the same methodology^(^^17^^–^^20^^)^ and formed the basis for the interpretation of these results.

Considering the importance of body musculature on glucose metabolism and the existence of metabolic and endocrine alteration in some muscular dystrophies, the present study aimed to evaluate parameters related to carbohydrate metabolism in golden retriever dogs that are carriers or affected by GRMD, since information in the literature is currently unavailable.

## Experimental methods

All procedures were approved by the Ethics and Animal Welfare Committee (protocol no. 13.1.1505.74.7) of the College of Animal Science and Food Engineering, University of São Paulo.

### Animals and experimental design

The study was performed at a kennel located at the Department of Anatomy of Domestic and Wild Animals of the College of Veterinary Medicine and Animal Science at the University of São Paulo.

The dogs were chosen according to the kennel availability and randomised into three experimental groups: healthy/control (G1), carrier GRMD females (G2) and dogs affected by GRMD (G3), totalling six animals per experimental group.

The affected and carrier animals underwent a periodic clinical evaluation every 15 d and were weighed once per week. The groups of carrier females and controls presented ideal body condition score (4 or 5) whereas affected dog scores were under or equal to 4, according to Laflamme^(^^21^^)^.

The classification of the GRMD carriers or affected animals was confirmed by performing biochemical, immunohistochemical and molecular diagnostic methods, which consisted of detecting a high concentration of the creatine kinase isoenzyme and a lack of dystrophin protein^(^^22^^,^^23^^)^ at the moment of birth by collecting blood samples from the umbilical cord. The tests were repeated for confirmation after 30 d.

### Diet

During the study, all animals received the same commercial dry diet (Royal Canin Medium Junior; Royal Canin Brazil), and were allowed 7 d for adaptation to the diet before the start of data collection. The amount of food offered to each animal was calculated according to National Research Council^(^^24^^)^ recommendations, using the equation: 95 × (body weight)^0·75^ = kcal/d (397 × (body weight)^0·75^ = kJ/d). The food was ground and hydrated with warm water and offered as a paste for all animals as a way to standardise the management employed for the affected animals due to their difficulty in seizing and swallowing.

### Postprandial response test

These tests were performed after 12 h of fasting and each dog was aseptically catheterised using a peripheral intravenous catheter (Becton Dickson). Blood samples were taken before feeding (baseline sample, time 0) and 5, 10, 15, 30, 60, 120, 180, 240, 300 and 360 min after feeding according to Carciofi *et al.*^(^^25^^)^ and Brunetto *et al.*^(^^26^^)^. Samples destined for glucose analysis were collected in fluoridated EDTA tubes, and samples for fructosamine and insulin analysis were collected in tubes without anticoagulant. Fructosamine was analysed only at time 0. All samples were immediately centrifuged and frozen until use.

### Laboratory analysis

Plasma glucose concentrations were determined by the glucose oxidase test (Glucose assay; Randox Laboratories Ltd), using semi-automatic glucose equipment (Randox Laboratories Ltd). Plasma fructosamine concentrations were determined by the kinetic method of fixed time (Frutosamina test; Labtest Diagnóstica S.A.) in the Multiuser Laboratory of the College of Veterinary Clinics of Food Engineering and Animal Science at the University of São Paulo. Insulin analysis was performed using human insulin containing ^125^I as a tracer hormone and 100 % of specificity for canine insulin (Human Insulin Specific; Millipore Corporation) at Genese laboratory (São Paulo, Brazil).

### Calculations and statistical analysis

The glucose increments were calculated by subtracting the baseline value for each animal from the values obtained during the 360 min of collections. The AUC were calculated for the glucose concentrations in the total range, which represents the 360 min of collections, and also for the following ranges: 0 to 60 min (AUC 0–60); from 0 to 120 min (AUC 0–120); from 0 to 240 min (AUC 0–240); from 60 to 120 min (AUC 60–120); from 60 to 240 min (AUC 60–240), and 60 to 360 min (AUC 60–360) after food consumption. Those areas were calculated using the mean blood glucose values at each time for all animals in each group, and AUC were obtained for G1, G2 and G3 by the trapezoidal numerical integration method. The results were analysed using GraphPad Prism (GraphPad Software)^(^^27^^)^. Evaluation of insulin sensitivity was calculated by the HOMA (homeostasis model assessment) index which takes into account the paired basal insulin with basal glucose levels, according to the equation: HOMA score = (basal serum insulin (mU/l) × (basal plasma glucose (mg/dl))/405 ((basal serum insulin (mU/l) × (basal plasma glucose (mmol/l))/22·5)^(^^19^^)^, and animals are considered insulin resistant for HOMA values higher than 2·4.

The obtained results were analysed by Statistical Analysis System software (SAS Institute Inc.)^(^^28^^)^, and were previously checked for normality of residues by the Shapiro–Wilk test and the variances were compared by an *F* test. Basal, minimum, mean and maximum glucose, basal insulin and fructosamine, HOMA index and ages were analysed using PROC GLM and experimental groups were compared by a Tukey test. For assessing postprandial response, statistical analyses were conducted in PROC MIXED with the definition of the matrices used based on the value of the Akaike information criterion, considering effects of group, time and interaction between groups and time as fixed effects and animal within the group as a random effect. The significance level was set at *P* < 0·05.

## Results

It was observed that plasma glucose concentrations at baseline (*P* = 0·0047), minimum (*P* < 0·0001), and mean (*P* = 0·0002) were higher in G2 and G3 than G1. Similarly, maximum glucose concentrations (*P* = 0·0359) in G3 were higher than those in G1. Baseline insulin concentrations presented an opposite trend, with G3 having reduced insulin concentrations compared with G1 (*P* = 0·0065). The HOMA index (*P* = 0·0298) was different between groups, with higher values in G1, and the fructosamine (*P* = 0·0160) was different only between G2 and G3, as presented in Table 1.

**Table 1.** Basal glucose, basal insulin, baseline fructosamine, homeostasis model assessment (HOMA) index, minimum glucose, mean blood glucose of all collection times and glucose maximum of the three experimental groups (Mean values with their standard errors)

	Groups						
	G1		G2		G3		
	Mean	se	Mean	se	Mean	se	*P*
Ages (years)	3·6	0·42	2·8	0·48	4·8	0·6	
Weight (kg)	32·17	0·91	29·37	1·42	19·53	1·15	
Basal glucose (mg/dl)*	73·02^b^	2·90	83·41^a^	1·33	88·06^a^	1·81	0·0047
Basal insulin (μU/ml)†	20·34^a^	3·79	12·52^a,b^	1·59	6·47^b^	0·98	0·0065
Baseline fructosamine (μmol/l)	259·54^a^	9·89	236·36^b^	3·99	270·17^a^	9·89	0·0160
HOMA index	2·60^a^	0·03	2·56^a,b^	0·31	1·40^b^	0·21	0·0298
Minimum glucose (mg/dl)*	63·18^b^	1·58	76·23^a^	1·43	78·19^a^	0·85	<0·0001
Mean blood glucose (mg/dl)*‡	79·42^b^	1·22	87·63^a^	0·88	87·90^a^	0·68	0·0020
Maximum blood glucose (mg/dl)*	91·92^b^	3·98	99·12^a,b^	1·22	100·26^a^	1·16	0·0359

G1, healthy/control group; G2, carrier females; G3, affected dogs.

^a,b^ Mean values within a row with unlike superscript letters were significantly different (*P* < 0·05; Tukey's test and ANOVA).

* To convert glucose in mg/dl to mmol/l, multiply by 0·0555.

† To convert insulin in μU/ml to pmol/l, multiply by 6·945.

‡ Mean blood glucose of all collection times.

**Fig. 1.**
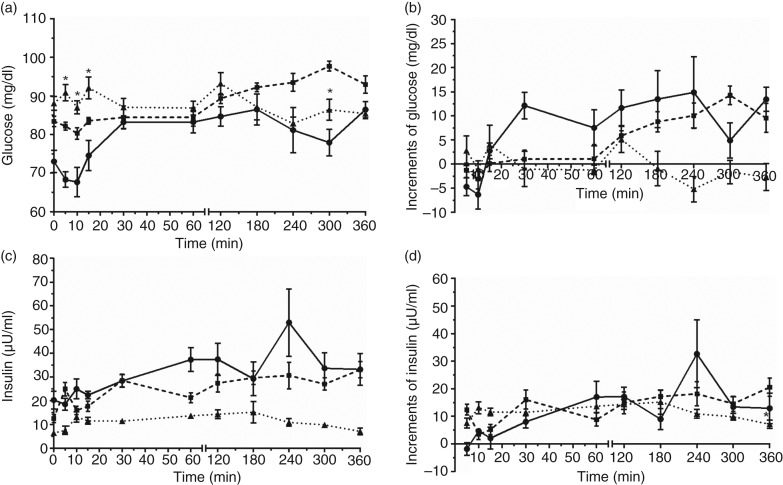
Values of blood glucose (a), insulin (c) and their respective increments (b, d) obtained by postprandial response tests of the healthy/control group (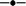), carrier females (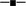) and affected dogs (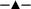). Values are means with their standard errors represented by vertical bars. * Mean value was significantly different from those of control and carrier dogs (*P* < 0·05; Tukey's test and ANOVA). To convert glucose in mg/dl to mmol/l, multiply by 0·0555. To convert insulin in μU/ml to pmol/l, multiply by 6·945.

With regards to postprandial glucose responses, an interaction was observed between time of collection and the experimental groups on the glycaemic curve (*P* = 0·0007), with fluctuations over time in G1 (5-min difference between 60, 120, 180 and 360 min, and 10-min difference between 180 and 360 min) and in minor proportions on G2 (10-min difference in 300 min). A similar behaviour was observed on glucose increment analysis (*P* = 0·0006). When glycaemic curves of the experimental groups were compared over time, higher glucose concentrations were observed in G3 than those in G1 at 5, 10 and 15 min (Fig. 1(a) and Table 1).

In relation to postprandial insulin responses, an interaction was observed between time and experimental group (*P* = 0·0149), with similar results of glucose: fluctuations of insulin concentration were seen in G1 (5-min difference in 60 and 120 min) and G2 (baseline difference from 5, 30 and 360 min), but no difference was seen over time for G3 dogs. The insulin increment also differed over time in G1 and G2, but remained constant in G3 (*P* = 0·0073). The insulinaemic curves of the experimental groups did not differ, whereas the insulin increment values observed in G2 were higher than those in G3 at 360 min (Fig. 1(c) and (d); Table 1).

Finally, in respect to age, no difference was found between G1 and G3 (*P* = 0·1431) and G1 *v.* G2 (*P* = 0·22). However, age was different between G2 and G3 animals (*P* = 0·0262).

## Discussion

GRMD dogs and carrier females presented higher basal plasma glucose concentrations compared with dogs in the control group, as well as minimum, average and maximum glucose levels. Following these results, regarding GRMD dogs, the observed higher plasma fructosamine values collaborate with the conclusion that this specific glycaemic behaviour represents not only the moment of collection, but apparently a recurrent behaviour. Nevertheless, all values remained within the reference range of normality (225–365 µmol/l) described by Nelson^(^^29^^)^.

Many factors^(^^30^^–^^36^^)^ may induce variations in insulin binding. In DMD patients, we found no relationship in the literature between insulin binding and serum insulin, steroid (sexual or adrenal) or creatine phosphokinase levels; furthermore, we found no circulating antibodies, such as insulin or receptor antibodies, to explain the reduced receptor concentration^(^^33^^)^. Thus, the cause of the impairment remains obscure. One possibility is that decreased receptor number is influenced by genetics^(^^35^^)^.

Freidenberg & Olefsky^(^^37^^)^ conducted oral glucose tolerance tests and insulin binding studies on erythrocytes from seventeen DMD and eight normal human males. Furthermore, they measured insulin binding to erythrocytes from twelve normal males and from eleven mothers and ten sisters of affected males. DMD patients had mild glucose intolerance and both fasting and postabsorptive marked hyperinsulinaemia (insulin resistance). Levels of glucose and insulin, expressed as incremental areas under their respective curves, were significantly elevated in the wheelchair-ridden patients. All of the ambulatory DMD males had normal oral glucose tolerance test results. Insulin binding to erythrocytes was 20–30 % lower in all DMD human patients than in normal males appropriately matched for age and degree of sexual development. This difference in binding was a result of lower affinity for the insulin receptor in DMD erythrocytes. On the contrary, insulin binding to fibroblasts was the same in normal males and DMD patients, suggesting that the abnormality of erythrocyte binding in DMD is probably not genetically induced.

Letellier *et al.*^(^^38^^)^ concluded that fasting insulin concentration and HOMA might provide quantifiable indices of disease progression. Rodríguez-Cruz *et al.*^(^^39^^)^ observed significant differences in insulin concentrations and HOMA-insulin resistance (IR) values among nutritional status groups, but no differences in age were detected. Overweight/obese boys presented higher glucose, insulin and HOMA-IR values compared with boys with normal BMI. Interestingly, an important percentage of underweight patients presented hyperinsulinaemia and IR (27·3 %). Body fat mass was higher in overweight/obese patients and lower in underweight patients than in patients with normal nutritional status. Both fasting insulin and HOMA-IR were positively correlated with all anthropometric and body composition parameters. However, the highest correlation was found between HOMA-IR and body fat mass.

Some hypotheses have been raised by some authors. Insulin is a powerful inhibitor of fat lysis and its deficiency is accompanied by lipolysis^(^^40^^)^. Importantly, the GRMD dogs show less deposition of body fat in addition to their muscular phenotypes. This can be explained partially by the decreased insulin concentrations in affected GRMD dogs, whereas another study from our research group found no differences in digestibility of nutrients in these animals^(^^41^^)^. The insulin receptors are located in the target cells of tissues such as liver, adipose tissue, and muscle cell membranes^(^^42^^)^. Some authors reported that muscle cell membranes from individuals affected by muscular dystrophy show progressive degeneration^(^^22^^)^ followed by replacement by fibrous and fat tissue^(^^43^^)^. It could be interesting to investigate if those changes in the plasma membranes of the myocytes could possibly lead to a decreased number of insulin receptors and, therefore, alter carbohydrate metabolism.

In this study, minimum serum concentrations of insulin were observed, which may have more relevance for the hepatocytes compared with glucagon. A decline in the insulin:glucagon ratio can induce increased gluconeogenesis and ketogenic capacity of hepatocytes^(^^40^^,^^44^^,^^45^^)^. The activation of gluconeogenesis is disadvantageous since it requires consumption of amino acids that could have been used for tissue synthesis and cell renovation. Instead, these are redirected to the formation of glucose and energy production; consequently, synthesis of this tissue is impaired, which can further aggravate the loss of muscle mass in animals that already have a reduced amount of this tissue.

In this respect, Grando *et al.*^(^^46^^)^ reported hepatomegaly associated with fatty infiltration after conducting ultrasounds on the abdominal region of dogs affected by GRMD. The authors attributed these findings to excess metabolites derived from the catabolism of skeletal muscle. The results of our study may partially explain the findings reported by these authors. The reduced production of insulin and metabolic adaptations exhibited by these animals for maintenance of glucose homeostasis can partially explain the muscle catabolism, fatty infiltration and increased liver size. Note that this information has not been previously reported in the literature. Combining the results obtained by the present study and previous studies, we can hypothesise that there is fatty infiltration of the liver as a compensatory response to low insulin secretion. Possibly, the GMRD dogs may have increased protein turnover due to higher accumulation of fatty acids in the liver by activation of lipolysis and also by increased gluconeogenesis.

Regarding age, according to the National Research Council^(^^24^^)^, there is no consensus about the influence of this parameter on carbohydrate metabolism. Strasser *et al.*^(^^47^^)^ have already described a lower glucose tolerance resulting from lower tissue insulin responsiveness in geriatric compared with young adult dogs. However, Lowseth *et al.*^(^^48^^)^ found no significant changes in blood glucose concentrations in beagle dogs aged 3 to 14 years.

At the same time, still according to the National Research Council^(^^24^^)^, geriatric dogs are considered to be, at least, 7 years old for medium breeds. In our study, the maximum age of affected dogs was 6 years old (only two dogs), which means that concerns related to variations of metabolism between G2 and G3 (where there is difference between ages) do not apply to the present experiment.

Nishio *et al.*^(^^49^^)^ observed in a correlational study that glucose concentration decreased with age and decrease in creatine kinase activity, i.e. with the progress of DMD. The NEFA and ketone body concentrations increased with decreasing glucose concentration.

The authors explain that muscle degeneration may result in depletion of substrates for gluconeogenesis. In the present study, however, the affected animals presented higher values of glucose compared with carriers and control animals. It is important to remember that the children studied by Nishio *et al*.^(49)^ were, in the majority, wheelchair-ridden and only two of them were able to walk with difficulty while our dogs, in the majority (four individuals), were still able to walk with difficulty. This difference in the ability to perform muscle activity and progression of disease may justify such variations.

All animals in this study showed normal HOMA index values according to the study of Xenoulis^(^^19^^)^. Therefore, insulin resistance was not detected in any of the groups.

### Conclusion

In conclusion, GRMD animals have changes in carbohydrate metabolism, with lower basal insulin and higher glucose in plasma compared with carriers and control animals, but do not show insulin resistance.
